# Behavioral Approaches to Study Top-Down Influences on Active Listening

**DOI:** 10.3389/fnins.2021.666627

**Published:** 2021-07-09

**Authors:** Kameron K. Clayton, Meenakshi M. Asokan, Yurika Watanabe, Kenneth E. Hancock, Daniel B. Polley

**Affiliations:** ^1^Eaton-Peabody Laboratories, Massachusetts Eye and Ear, Boston, MA, United States; ^2^Department of Otolaryngology – Head and Neck Surgery, Harvard Medical School, Boston, MA, United States

**Keywords:** temporal expectation, top-down, active listening, corticofugal, auditory streaming, descending, efferent, signal detection theory

## Abstract

The massive network of descending corticofugal projections has been long-recognized by anatomists, but their functional contributions to sound processing and auditory-guided behaviors remain a mystery. Most efforts to characterize the auditory corticofugal system have been inductive; wherein function is inferred from a few studies employing a wide range of methods to manipulate varying limbs of the descending system in a variety of species and preparations. An alternative approach, which we focus on here, is to first establish auditory-guided behaviors that reflect the contribution of top-down influences on auditory perception. To this end, we postulate that auditory corticofugal systems may contribute to active listening behaviors in which the timing of bottom-up sound cues can be predicted from top-down signals arising from cross-modal cues, temporal integration, or self-initiated movements. Here, we describe a behavioral framework for investigating how auditory perceptual performance is enhanced when subjects can anticipate the timing of upcoming target sounds. Our first paradigm, studied both in human subjects and mice, reports species-specific differences in visually cued expectation of sound onset in a signal-in-noise detection task. A second paradigm performed in mice reveals the benefits of temporal regularity as a perceptual grouping cue when detecting repeating target tones in complex background noise. A final behavioral approach demonstrates significant improvements in frequency discrimination threshold and perceptual sensitivity when auditory targets are presented at a predictable temporal interval following motor self-initiation of the trial. Collectively, these three behavioral approaches identify paradigms to study top-down influences on sound perception that are amenable to head-fixed preparations in genetically tractable animals, where it is possible to monitor and manipulate particular nodes of the descending auditory pathway with unparalleled precision.

## Introduction

During active listening, sound features that are distracting, irrelevant, or totally predictable are often suppressed and do not rise to perceptual awareness (Atiani et al., 2009; Galindo-Leon et al., 2009; O’Connell et al., 2011; Lakatos et al., 2013; Shepard et al., 2016; Sohoglu and Chait, 2016; Southwell et al., 2017). By contrast, anticipated changes in sensory inputs that guide perceptual decision making are often amplified (Fritz et al., 2003; Gutschalk et al., 2008; Lakatos et al., 2008; Mesgarani and Chang, 2012; Wiegand and Gutschalk, 2012; Atiani et al., 2014; Tsunada et al., 2016; Runyan et al., 2017). For example, a cue that precedes a target stimulus by a fixed temporal interval can provide a salient temporal expectation cue (Nobre and Van Ede, 2018). In active listening paradigms, temporal expectation can increase the probability of sound detection by as much as 40%, a robust effect that has been documented in species ranging from rodents to humans (Wright and Fitzgerald, 2004; Jaramillo and Zador, 2011; Buran et al., 2014; Carcea et al., 2017). The cell types and interconnected circuits that support enhanced processing of anticipated sounds are unknown but is hypothesized to have three essential properties: (i) It would have access to internally generated signals related to timing or preparatory motor actions, (ii) It would connect to lower-level stages of auditory processing to modify the gain and tuning precision of neurons that encode or compute anticipated sound features, (iii) The firing patterns of neurons within this circuit would have to change *before* the onset of an expected sound, to support enhanced processing and perceptual awareness of the subsequent target signal as it arrives.

The massive network of deep layer (L) auditory corticofugal projection neurons fulfill each of the requirements listed above and are therefore a prime candidate for supporting temporally cued active listening. Their apical dendrites reside in superficial cortical layers, where they likely intermingle with inputs from frontal cortex that encode the cue or timing-related inputs initiated by the cue (Xu et al., 2012; Zhang et al., 2014; Takahashi et al., 2016). The axons of deep layer corticofugal neurons innervate subcortical central auditory targets including the medial geniculate body, inferior colliculus, superior olivary complex, and dorsal cochlear nucleus (Diamond et al., 1969; Suga and Ma, 2003; Winer, 2005; Stebbings et al., 2014). As for the final requirement, a recent study from our lab discovered that layer L6 corticothalamic neurons, the largest component of the auditory corticofugal pathway, begin spiking hundreds of milliseconds prior to movements that trigger sounds and rewards (Clayton et al., 2021).

**FIGURE 1:**
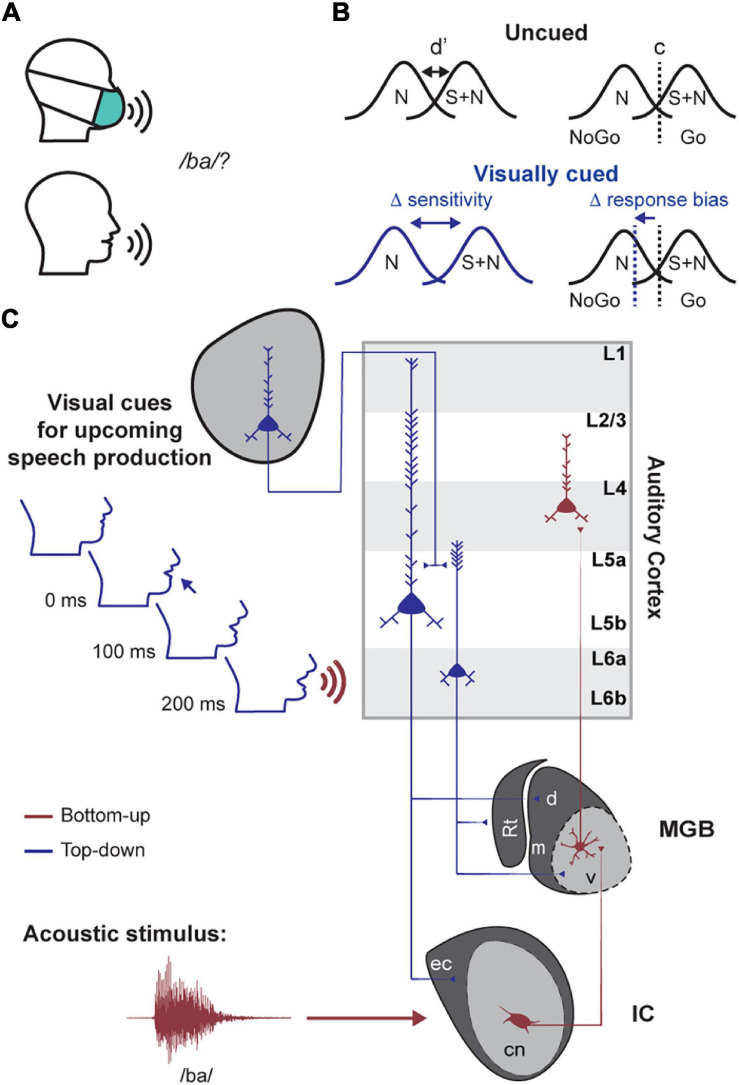
A proposed role for descending projections in predictive listening tasks. **(A)** A basic consonant recognition task is easier in the presence of visual speech cues (e.g., mouth movements). **(B)** Hypothesized changes in signal detection theory metrics between visually cued and uncued conditions. N = noise distribution; S + N = signal and noise response distribution. D-prime (d′) and c refer to the sensitivity and criterion terms, respectively. **(C)** Schematized circuit diagram for one way that cross-modal inputs elicited by facial movement could activate auditory corticofugal neurons for enhanced processing of expected speech cues. Top-down descending auditory corticofugal projections to the inferior colliculus (IC) and medial geniculate body (MGB) are illustrated in blue. Bottom-up corticopetal pathways are illustrated in red. Rt = thalamic reticular nucleus; cn/ec = central nucleus and external cortex of the IC; m/d/v = medial, dorsal and ventral subdivisions of the MGB.

As an example of how corticofugal neurons could mediate perceptual benefits of valid temporal expectations, consider the role of visual cues in the detection of a speech utterance (Figure 1). The mouth opens hundreds of milliseconds before speech begins (Chandrasekaran et al., 2009). This visual cue could then be exploited by the auditory system to enhance the processing and intelligibility of a target speech signal amidst a background of competing noise sources (Sumby and Pollack, 1954; Calvert et al., 1997; Grant and Seitz, 2000). In this example and in other types of predictive listening, corticofugal neurons could be the nexus between long-range signals carrying predictive cues and subcortical circuits that process low-level sound features. This hypothesis and others like it could be tested in head-fixed studies of genetically tractable animal models, such as mice, which offer unique advantages over other model systems and freely moving preparations for performing targeted recordings and manipulations of specific types of auditory corticofugal neurons (Bajo et al., 2010; Xiong et al., 2015; Asokan et al., 2017; Guo et al., 2017; Williamson and Polley, 2019; Clayton et al., 2021). However, before launching into the neuroscience studies, a behavioral framework to study temporal expectation in head-fixed mice must first be established.

As a first step, this paper details three different operant behavioral approaches to studying predictive listening in head-fixed mice. Across all tasks, we used operant Go/No-Go behaviors that facilitated rapid acquisition of task performance and hundreds of trials per behavioral session, allowing comparison of psychometric functions across conditions where the timing of target stimuli is predictable or not. We interpreted our results through the lens of signal detection theory to tease apart whether expectation-related changes in perceptual thresholds were mediated by changes in the observer’s sensory sensitivity or decision criterion (Figure 1B; Green and Swets, 1966; Stanislaw and Todorov, 1999). We provide evidence that sensory and cognitive cues over multiple timescales enhance auditory perception, which provides a behavioral framework for future work that will monitor and manipulate corticofugal neurons during appropriate behaviors to identify their causal involvement in active listening.

## Materials and Methods

### Subjects

All procedures in mice were approved by the Animal Care and Use Committee of the Massachusetts Eye and Ear Infirmary and followed guidelines established by the NIH for the care and use of laboratory animals. A total of 29 mice of both sexes were used. All mice were 6-8 weeks old at the beginning of experiments.

All procedures in humans (*N* = 9, age range: 20-48, 2 females) were approved by the institutional review board at the Massachusetts Eye and Ear Infirmary. Eligibility of participants was determined by screening for cognitive function (Montreal Cognitive Assessment, MOCA > 25), depression (Beck’s depression inventory, BDI < 21 for inclusion), tinnitus (Tinnitus reaction questionnaire, TRQ < 72 for inclusion), use of assistive listening devices (subjects were excluded if they reported use of cochlear implants, hearing aids, bone-anchored hearing aids or FM assistive listening devices) and familiarity with English (subjects were excluded if they did not report at least functional fluency). Normal hearing was confirmed in eligible participants by measuring audiometric thresholds in a double walled acoustic chamber (≤20 dB HL for frequencies up to 8 kHz).

### Visually Cued Tone-in-Noise Detection in Human Subjects

Human subject testing occurred in a single-walled walk-in chamber under ambient illumination. Subjects were seated 1 meter in front of a 3 mm red LED and held a response button to indicate detection of the target sound. Sound stimuli were delivered diotically using calibrated headphones (Bose AE2). Digital and analog signals controlling acoustic and visual stimulus presentation were controlled by a PXI system with custom software programmed in LabView (National Instruments). Subjects each completed one 90-min testing session.

In the Go/No-Go tone-in-noise detection task, subjects were required to detect a 12 kHz tone burst (100 ms duration with 5 ms cosine-square onset/offset ramps) of varying intensity in the presence of continuous 50 dB SPL white noise. Subjects indicated detection by button pressing within 2 s following sound onset. Auditory feedback on correct detections was provided with a positive-valence speech token (“Yay!”). No explicit feedback was provided for any other trial outcome (miss, correct reject, or false alarm), but button presses that occurred during catch trials where no tone was presented were followed by a timeout of 5 s. Each trial was followed by an 8-12 s intertrial interval (ITI) drawn from an exponential distribution to maintain a flat hazard rate.

To probe visually cued expectations, we used a 3.5 s visual cue which ramped in brightness in each trial before instantaneously terminating 2 s before tone onset. Performance was compared between interleaved visually cued and uncued trials. To familiarize subjects with the visually cued contingency and determine baseline thresholds, we began by varying target tone level (starting intensity: 40 dB SPL, step size: 1 dB) using a one-up, one-down adaptive procedure with six reversals to determine the subjects’ 50% correct thresholds (Levitt, 1971). This adaptive procedure was repeated twice and averaged to determine each subject’s detection threshold. In testing blocks (8 blocks of 22 trials each), tone levels for each subject were set relative to the adaptive threshold (−7.5 to 7.5 dB SPL re: threshold in steps of 2.5 dB SPL), with additional catch trials in which no target stimulus was presented. In the test blocks, 33% of trials were visually cued. Tone level and visual cue presentation were randomized across trials.

### Preparation for Head-Fixed Mouse Behavior

Prior to behavioral training, mice were implanted with a headplate for subsequent head-fixation during behavioral sessions. Briefly, mice were anesthetized with isoflurane (Piramal) in oxygen (5% induction, 2% maintenance). Lidocaine hydrochloride was administered subcutaneously to numb tissue overlaying the dorsal surface of the skull. The skull was then exposed by retracting the scalp and removing the periosteum. Prior to headplate placement, the skull surface was prepared with etchant (C&B metabond) and 70% ethanol. A custom headplate (iMaterialize, Romero et al., 2020) was then affixed to the skull using dental cement (C&B metabond). Following surgery, Buprenex (0.05 mg/kg) and meloxicam (0.1 mg/kg) were administered and the animal was transferred to a heated recovery chamber.

From 3 to 5 days after the headplate surgery, animals were placed on a water restriction schedule (1 mL/day). Behavioral training began when animals reached a target weight of 80% of their initial body weight. Throughout behavioral training, animals were weighed daily and monitored for signs of dehydration. If mice did not receive 1 mL during a given training session, they were provided with supplemental water. Behavioral sessions took place in dimly lit, single-walled sound-attenuating booths (Acoustic Systems and Med Associates), where mice were placed on an electrically conductive cradle and head-fixed. For tone detection tasks, a single lick spout was positioned 1 cm from the animal’s mouth using a 3D micromanipulator (Thorlabs). For the self-initiated frequency recognition task, an apparatus consisting of two lickspouts (4 cm apart) was positioned 1 cm below and 0.5 cm to the right of the animal’s snout. Lick spout contact was registered by an electrical circuit which produced a 5 V output signal whenever the tongue closed the circuit between lickspout and the cradle. Lick spout signals were digitized at 400 Hz. Freefield acoustic stimuli were presented through an inverted dome tweeter positioned 10 cm from the animal’s left ear (CUI, CMS0201KLX). A second inverted dome tweeter was placed below the first tweeter at the same distance and azimuthal position for presentation of continuous white noise. Speakers were calibrated before behavioral training using an ultrasonic acoustic sensor (Knowles Acoustics, model SPM0204UD5). For the visually cued tone detection and frequency recognition tasks, a broad spectrum LED (Thorlabs) was placed 20 cm away, in the left visual hemifield. As per human subjects testing, all stimuli, reward delivery, and behavioral contingencies were controlled by a PXI system with custom LabVIEW software.

### Behavioral Shaping and Testing in Head-Fixed Mice

#### Task 1: Light Cued Tone-in-Noise Detection

All mice were habituated to head-fixation for 1-2 sessions before beginning behavioral shaping. Shaping began by conditioning mice to lick for a 70 dB SPL target tone in the presence of 50 dB SPL white noise by presenting a small water reward (4-6 μL) 0.2 s after tone onset. Once licking began to precede reward delivery, animals were moved to an operant version of the task where they were required to lick between 0.2 and 2 s from tone onset to receive reward. Once operant hit rates exceeded 80%, we added additional target intensities to obtain full psychometric functions. Responses during catch trials in which the tone was not presented resulted in a 5 s time out. The visual cue was introduced once detection thresholds reached an asymptote and false alarms rates were consistently below 30% (∼15 sessions into shaping).

Each testing session began by obtaining a 50% detection threshold using a modified 1-up,1-down adaptive procedure (one track with six reversals, 70 dB SPL initial level, 5 dB initial step size, 2.5 dB SPL step size after the first reversal). Catch trials (50% probability) were randomly interleaved in the adaptive track to determine whether psychophysical performance was under stimulus control. Once we had estimated a 50% correct detection threshold, target tone intensities for testing blocks (36 trials, 4-10 blocks per session) spanned −5 dB to +5 dB re: threshold in 2.5 dB steps. In testing blocks, the visual cue was randomly presented in 33% of trials, with an identical waveform and time course as in the human version of the task. Every trial was followed by an 6-10 s ITI drawn from an exponential distribution to maintain a flat hazard rate. Mice performed 200-400 trials per day.

#### Task 2: Detection of Regular or Jittered Target Streams in a Tone Cloud Background

Mice were maintained on water restriction and adapted to head restraint, as described above. Shaping for the tone-in-cloud detection task was similar to the tone in noise task. In this task, mice were required to detect a repeating 16 kHz target tone (13 individual bursts, each 20 ms in duration, 5 ms cosine-squared ramps) repeated every 480 ms (2.08 Hz) embedded in a continuous tone cloud background. The tone cloud consisted of serially presented tone bursts of varying frequency selected at random (4-48 kHz range of 0.08 octave spacing, 40 dB SPL, 20 ms burst duration, 5 ms onset/offset cosine-squared ramps, 50 Hz repetition rate). A one octave protected bandwidth was included around the target frequency to limit energetic masking (Micheyl et al., 2007; Yin et al., 2007). Each trial began with tone cloud presentation for between 3 and 6 s, randomly drawn from a truncated exponential distribution. If licking occurred in a 2 s window prior to target onset, the countdown to target presentation was extended by another 2 s. Hits were operationally defined as lick spout contact occurring no earlier than 200 ms after the onset of the first target burst and no later than 480 ms after the last burst. Initial conditioning was performed at a signal to noise ratio of 35 dB (75 dB SPL target level). Each trial was followed by an ITI of 4 s. Once animals showed hit rates > 90%, catch trials where no target was presented were introduced. False alarms resulted in a 10 s time out. When false alarm rates fell below 40%, additional target levels were introduced to obtain psychometric functions across a range of signal-to-noise ratios (SNR). Once Go probabilities across catch trials and the full range of SNRs demonstrated that performance was reliably under stimulus control, we introduced a condition where the 13 target tones were presented at a fixed SNR (30 dB) either periodically (at 2.08 Hz) or aperiodically. In the aperiodic condition, the onset timing of tone bursts 2-12 were independently jittered with a time interval selected at random (±20-220 ms). Mice performed 100-200 trials per day.

#### Task 3: Self-Initiated Frequency Recognition

Mice were maintained on water restriction and adapted to head restraint, as described above. During initial shaping, mice were conditioned to lick the trial initiation spout within 8 s of LED onset to receive a small quantity of water (2 μL). Once mice learned to initiate trials, they were then conditioned to lick the decision spout within 0.2-2 s after the 12 kHz target tone was presented, but not a 6.5 kHz non-target tone (1.5s after initiation, 0.1s tone duration with 5ms raised cosine onset/offset ramps at 70 dB SPL). Lick spout contact during the response window following a non-target (foil) tone resulted in a 4-5 s time out. Contact on the decision spout prior to tone onset ended the trial. Inter-trial intervals were drawn from an exponential distribution (3-10 s). Once mice learned to withhold licking on >80% of foil tones, additional foil frequencies were presented to measure a psychometric function. Once animals displayed <30% false alarm rates for the easiest foil frequency, we introduced blocks of computer-initiated trials where initiation spout licking did not trigger sound. Most mice required 1-2 weeks of behavioral shaping before they could perform the complete frequency recognition task. Daily sessions consisted of 2-5 blocks of self-initiated or computer-initiated trials. Blocks were pseudorandomly interleaved and consisted of 100 trials each. Foil tones were presented in 50% of trials and were randomly selected from five logarithmically spaced frequencies centered on the indecision point (50% false alarm rate) from the previous session. Of 126 behavioral sessions, 10 were excluded either because the mean hit rate was less than 80%, or fewer than 200 total trials were performed.

Once reliable psychometric functions were obtained for self- and computer-initiated trial conditions, we included an addition experiment condition in which the typical 1.5 s foreperiod between self-initiation and target onset was perturbed in a small fraction (5%) of trials. The particular set of altered foreperiods varied across mice to maximize coverage of a wide-range of delays (0.25 to 1.25 s following self-initiation, in.25 s steps). Violation of the expected foreperiod were always shorter – never longer – than the expected 1.5 s delay.

### Data Analysis

Psychometric functions were fit using binary logistic regression. Recognition thresholds (50% Go probability) were determined using the fit psychometric functions. D-prime was calculated as z(hit rate) – z(false alarm rate). The criterion *c* was calculated as – (z(hit rate) + z(false alarm rate))/2 (Stanislaw and Todorov, 1999). For the self-initiated frequency recognition task, d-prime and c values were averaged over all tested frequencies. Across all tasks, reaction time was calculated using the first lick latency on hit trials. Single trial reaction times less than 80 ms were considered artifactual and were not considered for further analysis. For the analysis of perturbations of the self-initiated foreperiod, we z-scored the reaction times from each session with respect to all reaction times for the expected foreperiod. This approach allowed us to compensate for overall shifts in reaction times across days due to changes in motivation, vigilance, or spout placement.

For all paired difference tests, the mean of each subject’s performance across sessions was compared between conditions, as each session from the same mouse could not be considered an independent measurement. Linear regression was used to test foreperiod perturbation effects as each animal was only presented with a pseudorandom subset of delays (mean = 3.25 of 5 possible delays) due to the large number of trials required to obtain psychometric functions for each sparsely presented delay. A linear mixed effects model was used to determine the relationship between frequency recognition thresholds and pre-stimulus licking, while accounting for mouse identity and session numbers as random effects.

## Results

### Experiment 1: A Predictive Visual Cue Enhances Sound Detection Thresholds in Humans but Not in Mice

**FIGURE 2:**
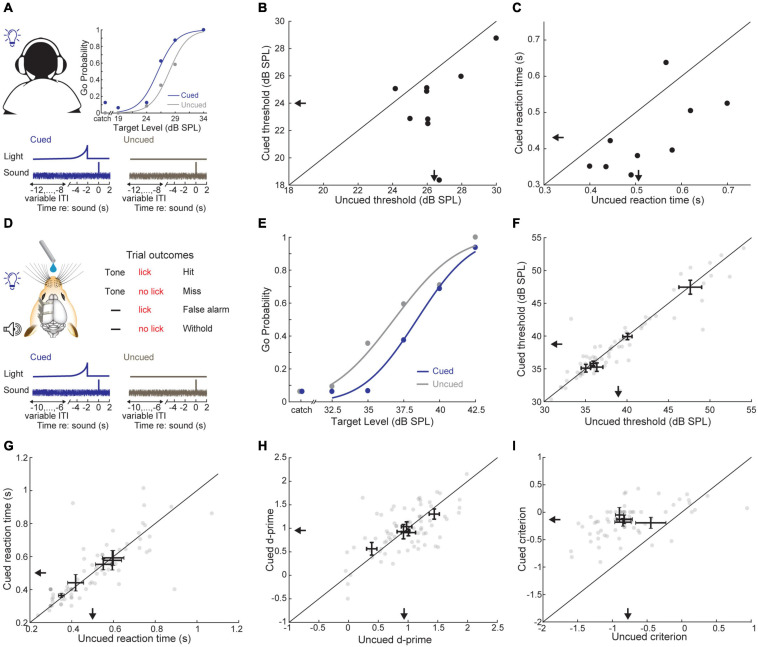
A predictive visual cue decreases tone-in-noise detection thresholds and reaction times in humans, but not mice. **(A)** Schematic of the visually cued tone-in-noise task in human listeners with example psychometric functions for one subject (top) and cue structure (bottom). ITI = inter-trial interval. **(B)** Visually cued and uncued 50% correct thresholds within a behavioral session. Each dot represents an individual subject. Arrows indicate group means. **(C)** Reaction times for visually cued and uncued hit trials. **(D)** Schematic of the visually cued tone-in-noise detection task in water-restricted, head-fixed mice. Trial structure was identical to the human task, except that mice received water rewards on hit trials. **(E)** Probability of a Go response as a function of tone intensity for a single representative behavioral session. Psychometric fits (lines) and actual performance (dots) are shown for visually cued (blue) and uncued trials (gray). In catch trials, the false alarm rate is determined by presenting the visual cue without the auditory target. **(F)** Visually cued and uncued 50% correct thresholds. Each gray dot represents a single behavioral session from one mouse. Bidirectional error bars show mean ± 1 S.E.M. visually cued and uncued thresholds for one mouse. Arrows indicate group means. **(G)** The sensitivity term, d-prime, for visually cued and uncued trials, plotted as in panel **(F)**. **(H)** The criterion term, c, for visually cued and uncued trials, plotted as in panel **(F)**. **(I)** Reaction time (i.e., time to first lick) for visually cued and uncued hit trials, plotted as in panel **(F)**.

To probe the role of predictive sensory cues in auditory perception, we devised a simple cued tone-in-noise detection task where an LED terminated 2 s before tone onset in 33% of trials (Figure 2A). First, we attempted to replicate previous studies showing that predictive visual cues enhance auditory detection in humans (Grant and Seitz, 2000) using a Go/No-Go task design that would easily translate to mice. Subjects (*N* = 9) indicated tone detection by pressing a button and were required to withhold from responding otherwise. We found that tone-in-noise detection thresholds were significantly lower when preceded by the visual cue (cued: 24.03 ± 0.96 dB SPL, uncued: 26.43 ± 0.57 dB SPL, Wilcoxon signed-rank test, *Z* = −2.54, *p* = 0.015; Figure 2B). Further, reaction times were shorter for visually cued trials (cued: 0.43 ± 0.03 s, uncued: 0.53 ± 0.03 s, signed-rank test, *Z* = −2.31, *p* = 0.02; Figure 2C), consistent with previous work on the role of predictive cueing in sound detection (Greenberg and Larkin, 1968; Wright and Fitzgerald, 2004; Best et al., 2007).

We trained mice in an operant detection task modeled after the conditions used above in human subjects (Figure 2D). Across both conditions, mice (*N* = 5 mice, *n* = 72 sessions) showed tone detection performance that was qualitatively similar to human observers, with low false alarm rates on catch trials and steeply sloping psychometric functions across a range of target tone intensities (Figure 2E). However, we did not find any consistent benefit for the visual cue on detection thresholds (cued: 38.71 ± 2.37 dB SPL, uncued: 39.00 ± 2.32 dB SPL, signed-rank test, *Z* = −1.75, *p* = 0.08; Figure 2F) or reaction times (cued: 0.50 ± 0.04 s, uncued: 0.50 ± 0.05 s, signed-rank test, *Z* = 0.40, *p* = 0.69; Figure 2G). To better understand if the weak effects of the visual cue on threshold and reaction time belied underlying changes in sensitivity or response bias, we turned to signal detection theory, which provides a means for formal assessment of both measures (Green and Swets, 1966; Stanislaw and Todorov, 1999). Visual cueing had little effect on the separability of signal and noise distributions, as measured by d-prime (cued: 0.95 ± 0.12 stds, uncued: 0.95 ± 0.17 stds, signed-rank test, *Z* = 0.13, *p* = 0.89; Figure 2H). By contrast, the response bias was significantly reduced (i.e., biased toward NoGo rather than Go responses) when target tones were preceded by a visual cue (cued: −0.14 ± 0.03 stds, uncued: −0.77 ± 0.08 stds, signed-rank test, *Z* = 2.02, *p* = 0.04; Figure 2I). These findings suggest that the visual cue was perceived by both species. Human subjects were able to exploit the visual predictive cue to more reliably perceive liminal tones in noise, whereas in mice, the visual cue altered their overall behavioral response bias without affecting their perceptual sensitivity to the target stimulus.

### Experiment 2: Mice Exploit Temporal Regularities to Perform an Auditory Streaming Task

**FIGURE 3:**
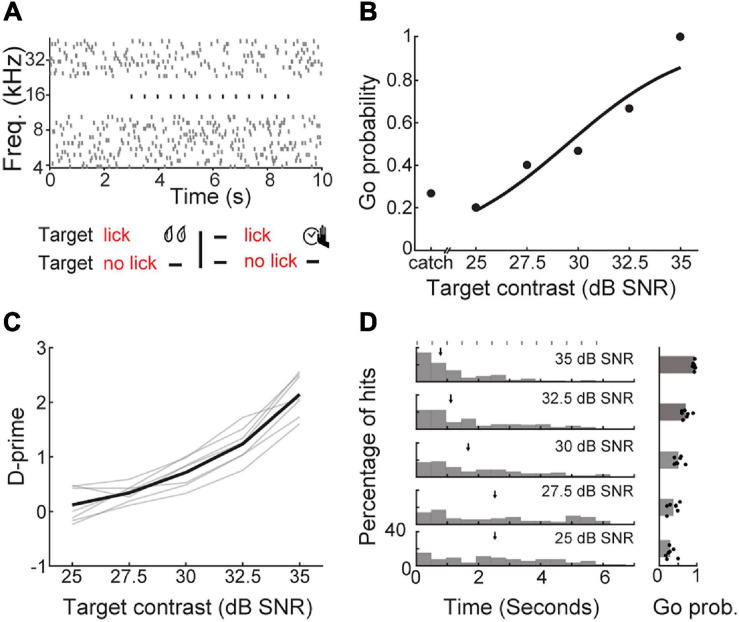
Detection of a periodic target in background noise. **(A)** Schematic of a Go/No-Go detection task that required mice to report the perception of a repeating target tone in the presence of a tone cloud background. Top; the stimulus spectrogram for a trial in which the repeated target tone (black dots) begins 3 s after the onset of the random, serially presented masking tones (gray dots). Bottom; task contingencies. **(B)** Go probability as a function of target contrast for a single representative session. **(C)** D-prime as a function of target contrast, showing the mean (black line) and individual mice (gray lines). **(D)** Left, reaction time distributions as a function of target contrast. Median reaction times are indicated with arrows. Target burst timing is indicated by gray rectangles. Right, Go probability as a function of target contrast, showing the mean and individual values (dots).

Interactions between visual predictive cues and auditory targets could be constrained by multi-sensory temporal binding windows derived from natural scene statistics or neural circuits subserving multisensory integration (van Wassenhove et al., 2007; Stevenson et al., 2011). In mice, the 2 s delay between visual cue offset and sound onset may have proved too long a gap for the visual stimulus offset to facilitate auditory detection (Siemann et al., 2015). To study the perceptual benefits of auditory temporal expectation in mice without relying on cross-modal integration, we turned to a within-modality cue. In auditory scene analysis, repetition provides a salient grouping cue for separating an auditory stream or object from background stimuli (Kidd et al., 1994; Gutschalk et al., 2008; Agus et al., 2010; Andreou et al., 2011; McDermott et al., 2011). In human listeners, presenting a repeated signal in the midst of an ongoing mixture produces a stream segregation phenomenon where the repeated signal pops out from the mixture after several repetitions (Micheyl et al., 2007; Gutschalk et al., 2008; McDermott et al., 2011). We tested whether a similar phenomenon existed in mice by tasking them with operantly reporting the presence of a regularly repeated target tone embedded within a tone cloud, with similar outcome contingencies as in the visual-cued tone detection task (Figure 3A).

To determine if mice could perform stream segregation using embedded repetition, we varied the sound level of the target tone relative to the tone cloud to obtain psychometric functions (*N* = 7 mice, *n* = 18 sessions; Figure 3B). As the signal to noise ratio (SNR) improved, d-prime increased (one-way repeated measures ANOVA, *F*(6, 24) = 152.17, *p* < 1 × 10^–15^; Figure 3C). At high SNRs, mice typically responded after the first target tone burst, suggesting that the intensity contrast between the target and the background was sufficient to support detection (Figure 3D). At less favorable SNRs, median reaction times occurred after two to three target tone bursts, consistent with the time course of build-up for repeating targets in human listeners. However, the longer reaction times we observed with decreasing SNR could also be attributed to the reduced stimulus intensity and not the regular repetition of the target, *per se* (Piéron, 1913; Viemeister and Wakefield, 1991).

**FIGURE 4:**
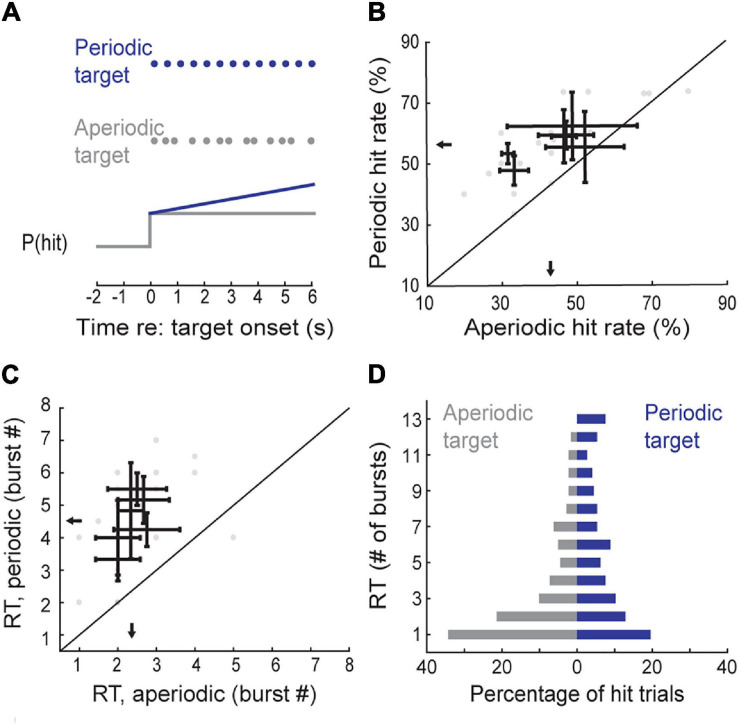
Temporal regularity enhances the perceptual salience of a repeated target. **(A)** *Top*, schematic of periodic and aperiodic target presentation. *Bottom*, hypothesized “build-up” of detection probability based on temporal regularity. **(B)** Hit rates for interleaved periodic and aperiodic targets presented at a fixed contrast (30 dB SNR). Data from individual sessions are shown as gray circles. Black lines represent the mean ± SEM hit rates for each mouse. Arrows indicate group means. **(C)** Reaction times, in number of bursts, plotted as in panel **(B)**. **(D)** Histograms of reaction times, pooled across all session and subjects, for periodic and aperiodic trains of target bursts.

To directly test whether long reaction times were a result of stream segregation based on rhythmic repetition, we jittered the repetition rate of the target tone at an intermediate SNR (30 dB), where reaction times suggested that target perception might benefit from temporal integration. We reasoned that if detection reflected a purely probabilistic process based on the stimulus contrast for each individual tone, presenting the targets aperiodically would not impact detection probability or reaction time distributions (Figure 4A). However, if target recognition benefited from the regular periodicity of the target, the likelihood of target detection would increase with repetitions of the target, and reaction times for regular targets would be skewed toward later repetitions.

Consistent with the latter hypothesis for temporal integration, mice were more likely to detect the target stream when it was regularly repeated (*N* = 6 mice, *n* = 19 sessions; periodic: 56.14 ± 2.10%, aperiodic: 43.34 ± 3.52%, signed-rank test, *Z* = 2.20 *p* = 0.03; Figure 4B). More to the point, median reaction times were significantly longer on hit trials in the periodic condition compared to the aperiodic condition (periodic: 4.51 bursts or 2.16 s, aperiodic: 2.37 bursts or 1.14 s, signed-rank test, *Z* = 2.21, *p* = 0.03; Figure 4C), suggesting that the improved overall detection probability with periodic target rates could be attributed to an increased probability of target detection later in the stream, which did not occur in the aperiodic condition. To determine if the distribution of reaction times between regular and jittered trials was different, we pooled data across all subjects and sessions. While the modal reaction time in hit trials corresponded to detection of the first tone in the target, over 34.67% of regular hits occurred after 6 bursts, compared to only 17.42% of the jittered hits (Kolmogorov-Smirnov two-tailed test, *D* = 0.23, *p* = 0.00004; Figure 4D).

### Experiment 3: Self-Initiation Enhances Frequency Discrimination

While each of the paradigms described above demonstrate the use of top-down cues in listening tasks, neither is optimized for future studies that combine behavioral assessments in head-fixed mice with neurophysiological methods to selectively monitor and manipulate auditory corticofugal neurons. For studies that will isolate the causal involvement of corticofugal neurons in temporal expectation, a behaviorally quiescent period between the cue and target sound, during which no explicit input is provided could prove useful for homing in on neural preparatory activity (Buran et al., 2014; Carcea et al., 2017). Further, if increased neural activity was observed prior to the onset of the target sound in either of the temporal expectation behaviors presented above, it would be difficult to disambiguate whether this activity reflected a neural representation of expectation or a motor preparatory signal related to the impending Go response (Clayton et al., 2021). This ambiguity could be resolved by task designs that either require subjects to make an overt behavioral report in all trials (e.g., alternative forced choice) or task designs where animals can make a correct decision by withholding a behavioral report.

**FIGURE 5:**
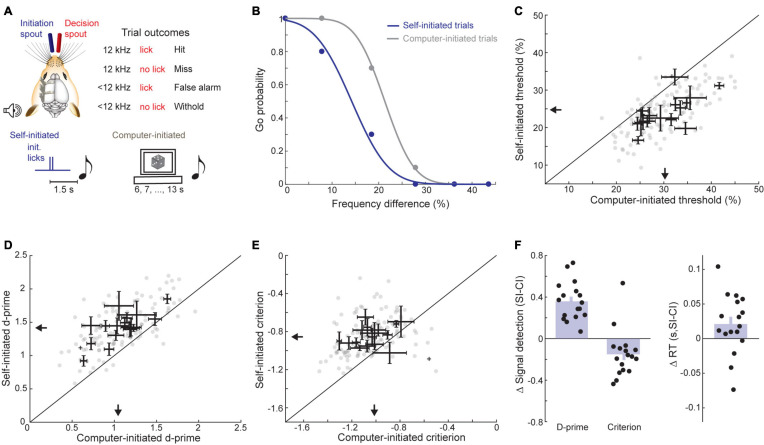
Trial self-initiation enhances frequency recognition thresholds. **(A)** Task design. *Top*, schematic of task set-up and contingencies for decision spout licking. *Bottom*, Target (12 kHz) and foil tones (<12 kHz) are presented with predictable timing following contact with the initiation spout (self-initiated, left) or with unpredictable timing determined by the computer (computer-initiated, right). **(B)** Representative psychometric functions from a single session with interleaved self- and computer-initiated trial blocks. **(C)** Self-initiated and computer-initiated 50% correct thresholds for frequency recognition. Each gray dot represents one behavioral session. Bidirectional error bars show mean ± 1 S.E.M. self-initiated and computer-initiated thresholds for each mouse. Arrows indicate group medians. **(D)** D-prime value, plotted as in panel **(C)**. **(E)** Criterion values, plotted as in panel **(C)**. **(F)** Quantification of mean changes in sensitivity, criterion, and reaction time between self-initiated and computer-initiated trials. Each black dot represents one subject and bars reflect the mean ± 1 SEM.

To address these limitations, we turned to a frequency recognition task where mice were trained to lick a decision spout following a 12 kHz tone, but withhold licking for tones of other frequencies (Figure 5A, top). To manipulate the availability of top-down information related to stimulus timing, target stimuli were occasionally presented at a fixed interval following self-initiated movement, rather than a cross-modal sensory cue, as it rules out the possibility that the preparatory cue was not detected (Reznik et al., 2014; Morillon et al., 2015). Trial self-initiation is a routine component of non-human primate and freely moving rodent behavioral tasks (Ghose and Maunsell, 2002; Nakajima et al., 2019). In freely moving rats and gerbils, sound detection and discrimination thresholds are better when animals self-initiate trials compared to conditions where sound presentation is unpredictable (Jaramillo and Zador, 2011; Buran et al., 2014; Carcea et al., 2017).

In our frequency recognition task, mice (*N* = 17 mice, *n* = 115 sessions) triggered sound 1.5 s later by licking a separate initiation spout to receive a small reward (Figure 5A, bottom). We contrasted blocks of self-initiated trials with blocks of computer-initiated trials where sound presentation timing was unrelated to contacts on the self-initiation spout. Psychophysical performance was under stimulus control in both conditions, as evidenced by high hit rates to the 12 kHz target and declining false positive rates at foil frequencies further away from the target (Figure 5B). Importantly, a clear top-down influence was observed in this behavior, as a clear reduction in target recognition threshold was observed across all mice and virtually all sessions in self-initiated trials (self-initiated: 24.61 ± 1.16%, computer-initiated: 30.35 ± 1.19%, sign-rank test, *Z* = −3.38, *p* = 0.0007, Figure 5C). Improved thresholds for self-initiated trials were mediated by an increased d-prime (self-initiated: 1.41 ± 0.06 stds, computer-initiated: 1.05 ± 0.07 stds, signed-rank test, *Z* = 3.62, *p* = 0.0003; Figures 5D,F) and a decreased bias to respond (self-initiated criterion: −0.86 ± 0.02 stds, computer-initiated: −1.01 ± 0.18 stds, signed-rank test, *Z* = 2.53, *p* = 0.01, Figures 5E,F). Contrary to previous studies in rats (Jaramillo and Zador, 2011; Carcea et al., 2017), we observed a tendency for slower reaction times on self-initiated trials (self-initiated: 0.24 ± 0.01 s, computer initiated: 0.22 ± 0.01 s; signed-rank test, *Z* = 1.96, *p* = 0.049; Figure 5F). Differences between this result and previous studies could be due to species differences or, more likely, to differences in the operant behavior (i.e., between freely moving nose-poke versus head-fixed licking).

### Sparse Violations of Temporal Expectations Reveal a Temporal Filter for Optimal Performance

**FIGURE 6:**
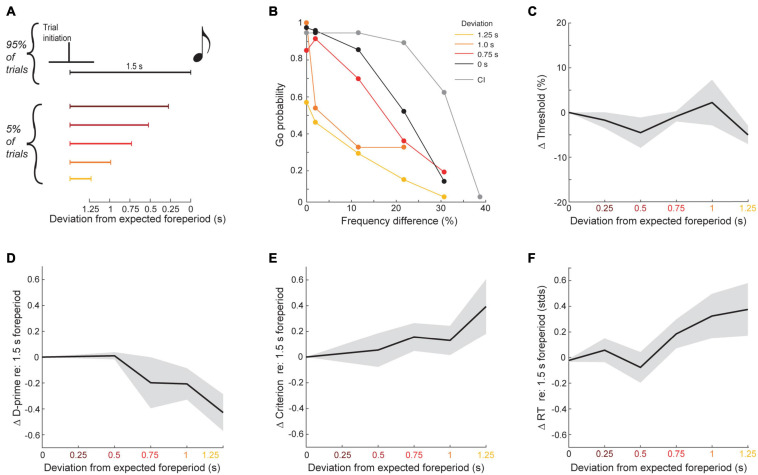
Frequency recognition is impaired when tones occur at unexpected times. **(A)** Schematic of foreperiod violations. **(B)** Go probability as a function of frequency difference for each tested delay in one example mouse. **(C)** Mean ± SEM change in thresholds as a function of deviation from the expected foreperiod across all mice. **(D)** Change in d-prime, as in panel **(C)**. **(E)** Change in criterion, as in panel **(C)**. **(F)** Change in z-scored reaction times, as in panel **(C)**.

The perceptual benefit of self-initiation could reflect the specific benefit of temporal expectations or simply a non-specific increase in arousal during blocks of self-initiated trials. To test whether the top-down benefit on self-initiated trials were consistent with a narrow window of increased temporal expectation or a more global elevation of arousal, we changed the foreperiod separating contact on the initial spout and sound onset from the typical 1.5 s duration to a shorter interval on a small fraction of trial (5%, *N* = 12 mice, *n* = 37,578 total trials over 200 sessions; Figure 6A). Violating the typical 1.5 s foreperiod by hundreds of milliseconds had striking effects on task performance, suggesting that self-initiation benefits reflected a specific time window of expected stimulus arrival. As shown in an example mouse, the typical self-initiation benefit seen on the majority of trials (black vs. gray lines in Figure 6B), was progressively changed when target and foil sounds were presented earlier than the expected timing. Importantly, the effect of foreperiod perturbations could not be seen in simple reports of frequency recognition threshold, on account of changes in both the hit and false positive rates (linear regression, *F*(1,48) = 1.29, *p* = 0.26, R^2^ = 0.03; Figure 6C). A signal detection theory analysis found that d-prime decreased as the sound onset deviated more from the expected timing, indicating that mice were less able to discriminate between target and foil at unexpected intervals (linear regression, *F*(1,48) = 6.74, *p* = 0.02, R^2^ = 0.21; Figure 6D). The criterion also became more positive, indicating that mice were less likely to respond at greater foreperiod violations and – based on the increased probability of misses to the 12 kHz target stimulus – may not have been perceptually aware that the stimulus was presented at all (linear regression, *F*(1,48) = 5.09, *p* = 0.03, R^2^ = 0.16; Figure 6E). Consistent with a specific temporal filter for listening, violations of the expected foreperiod duration were also associated with increased reaction times on Go trials (linear regression, *F*(1,48) = 7.29, *p* = 0.009, R^2^ = 0.13; Figure 6F).

### Frequency Discrimination Thresholds Are Influenced by How Animals Initiate Trials

**FIGURE 7:**
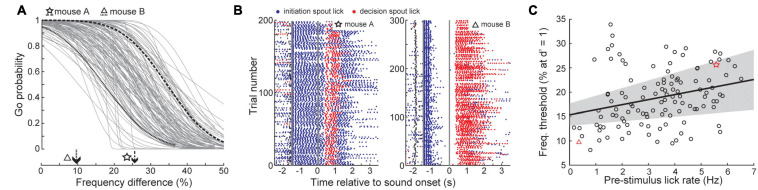
Vigorous licking during the foreperiod is associated with impaired frequency discrimination. **(A)** Psychometric functions for self-initiated frequency recognition trials across all behavioral sessions. **(B)** Lick rasters for the two example sessions shown in panel **(A)**. Blue and red dots denote lick timing on the initiation and decision spouts, respectively. Thick gray vertical lines indicate the timing of trial initiation and sound onset. Small vertical black lines indicate the timing of visual ready cue. **(C)** Frequency thresholds are improved in sessions with minimal licking prior to tone onset. Thick black line and gray shading represent the linear regression line and the bootstrapped 95% confidence interval of the linear regression, respectively. Each individual test session is represented by a dot, red symbols indicate example behavior sessions shown in panels **(A,B)**.

While reaction times were slower at unexpected intervals after self-initiation, it was surprising that self-initiated reaction times were 20 ms longer than for computer-initiated trials, as valid temporal expectations often speed decisions (Jaramillo and Zador, 2011). Further, we observed substantial heterogeneity in self-initiated thresholds across sessions (Figure 7A). To explain these outstanding sources of variability, we examined how mice performed the task at a more granular level by quantifying licking activity on the trial initiation spout. We noted that behavioral sessions with poor discrimination thresholds were associated with persistent licking of the initiation spout throughout the foreperiod (Figure 7B, left). In sessions with better discrimination thresholds, mice briefly licked the initiation spout and then paused before the target or non-target tone was presented (Figure 7B, right). Across all behavioral sessions, initiation spout lick rate during the foreperiod was negatively correlated with the discrimination threshold, even after controlling for other mitigating variables including the individual mouse and the number of prior testing sessions (linear mixed effects model, slope = −1.11% freq. diff/lick, *t*(117) = 2.69, *p* = 0.008; Figure 7C).

## Discussion

Knowing when to listen can enhance the detection of faint sounds or the discrimination of target sounds from distractors (Egan et al., 1961; Greenberg and Larkin, 1968; Wright and Fitzgerald, 2004; Best et al., 2007; Nobre and Van Ede, 2018). Here, we reported three inter-related behavioral experiments that explored how predictive sensory and motor cues enhance the perceptual detection and discrimination of upcoming sounds in humans and mice. Depending on the modality and timing of the predictive cue, valid expectations altered the observer’s perceptual sensitivity, response bias, or both. We found that a visual cue provided significant perceptual benefit to human listeners in a tone-in-noise detection task, but only a response criterion change in mice (Figure 2). A second experiment found periodicity aided the detectability of a liminal repeating target in a complex background noise by increasing the probability of late detection events, after the regularity of the target sound had been established (Figures 3, 4). In a third paradigm, we trained mice to expect a tone stimulus at a fixed interval after motor self-initiation, which results in significantly lower frequency recognition thresholds and improved sensitivity relative to unpredictable computer-initiated trials (Figure 5). The improved perceptual sensitivity for self-initiated sound was abolished when sound arrived approximately 1 s earlier than expected, suggesting a specific temporal window for enhanced auditory perception (Figure 6). A closer inspection of self-initiated frequency recognition trials revealed that exuberant motor activity during the foreperiod also interfered with frequency recognition accuracy, suggesting different types of internally generated signals that enhance or degrade perceptual performance (Figure 7).

Our approach to studying perceptual expectations in head-fixed mice was inspired by previous human psychophysical studies (Wright and Fitzgerald, 2004; Best et al., 2007). While head-fixed mice can perform two-alternative forced choice tasks (Sanders and Kepecs, 2012; Burgess et al., 2017; Vincis et al., 2020), we used Go/No-Go task designs which could be learned over the course of one to two weeks, allowing us to layer manipulations of perceptual expectations on top of basic detection or discrimination tasks (Guo et al., 2014; McGinley et al., 2015; Kuchibhotla et al., 2017). As appetitive Go/No-Go behaviors typically result in a bias toward responding (Gomez et al., 2007), the role of baseline response bias and motivational structure introduced by our task designs likely played a role in the expectation-related effects we observed. For instance, despite human listeners showing lower thresholds and faster responses in a visually cued tone-in-noise detection task, there was no effect of visual cueing on mouse detection thresholds or reaction times. Instead, we observed a shift in criterion in mice that led them to respond less frequently during visually cued trials across all sound levels. Similarly, in the self-initiated frequency recognition task, we observed decreased bias to respond during self-initiated trials, though the change in bias reported in this task were substantially smaller than the change in d-prime. The Go/No-Go tasks presented here have asymmetrical response requirements: while Go responses require just a single lick, No-Go responses require mice to withhold from licking throughout the entire response period. In this response structure, self-initiation or visual cueing could provide a warning signal to temporarily withhold from licking unless there is strong evidence that the target sound is present. Though the generality of our findings await testing in other operant behavioral task designs, our results demonstrate that predictive cues have varied effects on response bias and sensitivity that should be disambiguated by using signal detection theory or similar techniques.

Beyond the influence of task structure and reward contingencies, our results suggest that the effects of temporal expectations in head-fixed mice vary according to the modality and timing of predictive cues and the sounds they predict. In the visually cued task, the lack of changes in threshold or sensitivity we found might have been attributable to the relatively long delay between the visual cue and auditory target onset, as suggested from a recent report demonstrating improved tone in noise discrimination using a more proximal visual cue (400 ms foreperiod, Nakajima et al., 2019). Further, the spatial position of the visual cue could have contributed to our results in mice, a more ethologically relevant spatial position may have increased its behavioral salience (e.g., Yilmaz and Meister, 2013). Our second paradigm focused on intra-modal cues that can aid target perception on fast timescales. We found that SNR and temporal regularity provided independent bottom-up and top-down cues, respectively, to identify a target in a mixture. Our results in mice are consistent with previous studies in human listeners which collectively demonstrated that repetition is a salient grouping cue for auditory scene analysis (Kidd et al., 1994; Micheyl et al., 2007; Agus et al., 2010; Andreou et al., 2011; McDermott et al., 2011). The prolonged time course of repetition-based stream segregation suggests a mechanism by which repetitive inputs are integrated over time and used to predict the incoming acoustic signal. Our work provides behavioral proof-of-principle for future studies to uncover the neural substrates of this prolonged temporal integration process in a genetically tractable model organism.

Our third paradigm confirmed prior reports of improved thresholds and perceptual sensitivity when target sounds are presented at fixed time intervals following movement-based trial self-initiation (Jaramillo and Zador, 2011; Buran et al., 2014; Carcea et al., 2017). Improved discrimination in self-initiated trials could reflect differences in arousal or other global internal state differences (Rodgers and DeWeese, 2014; McGinley et al., 2015). By perturbing trial timing in a small fraction of trials, we showed that perceptual sensitivity decreased when sounds were presented at unexpected moments, arguing against purely arousal-mediated changes between self-initiated and computer-initiated trials. The pathways and processes underlying perceptual changes in self-initiated trials are less clear. Self-initiated movements can directly modulate central auditory processing, either through internal motor-corollary inputs or reafferent sensory inputs (Reznik et al., 2018; Schneider et al., 2018). Our findings in the self-initiated task suggest the combination of two opposing top-down influences on sound perception: on the one-hand temporal expectation clearly improved frequency recognition thresholds and perceptual sensitivity (Figure 5). At the same time, when movement-based contact on the trial initiation spout impinged too closely upon the sound delivery period, it adversely affected frequency recognition thresholds.

The generation of temporal expectations likely involves distributed circuits across frontal and parietal cortical areas, basal ganglia, hippocampus, and cerebellum (Janssen and Shadlen, 2005; MacDonald et al., 2013; Narain et al., 2018; Zhou et al., 2020). Many of the same regions implicated in temporal expectations are sensory responsive, but sound representations in these areas are context-dependent and evolve at slower timescales compared to representations within the early central auditory pathway (Rummell et al., 2016; Runyan et al., 2017; Elgueda et al., 2019). Our behavioral data show that top-down influences related to temporal expectations enhance even basic perceptual abilities like tone detection or recognition. Changes in basic auditory processing driven by top-down influences suggest a scheme where long-range inputs from brain areas involved in generating temporal expectations would modulate the fast-timescale, fine-grained encoding of acoustic features, which is generally restricted to the beginning of the central auditory pathway (Joris et al., 2004; Asokan et al., 2021).

While previous studies have reported changes in pre-stimulus neural activity during self-initiated auditory perceptual tasks, the precise neural circuits responsible for transforming long-range predictive inputs into changes in local network excitability based on temporal expectations and motor-corollary inputs remain relatively unexplored. Multiple lines of evidence from our lab suggest that a particular subclass of auditory corticofugal projection neuron, the layer 6 corticothalamic neuron (L6 CT), may play a central role in this process. First, artificial activation of auditory L6 CTs can enhance or impair sound discrimination, depending on the temporal delay between L6 CT spiking and sound presentation (Guo et al., 2017). At short delays between optogenetically induced L6 CT spiking and sound presentation (i.e., 100 ms), behavioral sound discrimination is enhanced, while at long delays (i.e., 200 ms), sound discrimination is impaired. Second, L6 CTs receive direct long-range inputs from motor-related areas such as the globus pallidus and increase their spiking hundreds of milliseconds before movements which predict sound and reward (Clayton et al., 2021). During trial self-initiation, L6 CTs would presumably be activated prior to contact with the initiation spout, shifting auditory cortex network excitability into an optimal state for discrimination, consistent with the improved behavioral discrimination we observed. However, any benefit derived from L6 CT activation would also depend on the precise alignment between L6 CT spiking and sound presentation, which may account for our observation that initiation spout licking which impinged on the sound delivery period impaired frequency recognition thresholds. While self-initiation is a strong predictive cue, future work which decouples movement-related activity in L6 CTs from cues that predict sound presentation timing could better elucidate how long-range predictive inputs sculpt L6 CT spiking to shift the auditory cortical network into an optimal state to process sounds at expected moments according to behavioral goals.

A role of auditory corticofugal cell-types in auditory processing and perception will likely require careful analysis of targeted corticofugal cell types in behaving animals. Although few studies have specifically investigated corticofugal contributions to sound-guided behaviors, their findings highlight a critical role of descending projections in contextual processing and experience-dependent plasticity (Bajo et al., 2010; Xiong et al., 2015; Guo et al., 2017; Homma et al., 2017). Seminal cell-type specific ablation studies found that auditory cortex neurons which project to the inferior colliculus play a key role in sensorimotor remapping after monaural deprivation (Bajo et al., 2010). Other work has shown that corticollicular neurons also control sound-driven innate defensive behaviors such as escape (Xiong et al., 2015). However, despite the potential role of descending projections in real-time subcortical gain control according to behavioral goals or attention, the necessary involvement of corticofugal neurons in these behaviors have yet to be demonstrated. With the development of predictive listening paradigms in head-fixed mice described here, a more complete understanding of how descending control in the auditory pathway guides adaptive behavior and active listening is within reach.

## Data Availability Statement

The raw data supporting the conclusions of this article will be made available by the authors, without undue reservation.

## Ethics Statement

The studies involving human participants were reviewed and approved by The Institutional Review Board at the Massachusetts Eye and Ear Infirmary. The patients/participants provided their written informed consent to participate in this study. The animal study was reviewed and approved by Animal Care and Use Committee of the Massachusetts Eye and Ear Infirmary.

## Author Contributions

KC, MA, and DP designed the study. KC, MA, and YW performed all the experiments and analyzed the data with supervisory input from DP. KH programmed the behavioral data acquisition software. KC and DP wrote the manuscript with feedback from all authors. All authors contributed to the article and approved the submitted version.

## Conflict of Interest

The authors declare that the research was conducted in the absence of any commercial or financial relationships that could be construed as a potential conflict of interest.
